# Supplementary material for: Living in the intertidal: desiccation and shading reduce seagrass growth, but high salinity or population of origin have no additional effect
Source: PeerJ. 2018 Jul 20;6:e5234. doi: 10.7717/peerj.5234 (PMC6055680; doi:10.7717/peerj.5234)
Supplement: Supplemental Information 2 — Std=standard deviation. [file peerj-06-5234-s002.docx]

**Supplementary Table 1**. Previously unpublished data of water column pH in salinity treatments described in van Katwijk et al. (1999), showing no differences in pH. Std=standard deviation.

| Raw data | | |  | Summary data | | |
| --- | --- | --- | --- | --- | --- | --- |
| replicate | salinity | ph |  | salinity | pH mean | pH std |
| 1 | 23 | 8.00 |  | 23 | 8.3 | 0.37 |
| 2 | 23 | 8.02 |  | 26 | 8.2 | 0.21 |
| 3 | 23 | 8.47 |  | 30 | 8.2 | 0.24 |
| 4 | 23 | 8.08 |  |  |  |  |
| 5 | 23 | 8.25 |  |  |  |  |
| 6 | 23 | 8.95 |  |  |  |  |
| 1 | 26 | 7.91 |  |  |  |  |
| 2 | 26 | 8.53 |  |  |  |  |
| 3 | 26 | 8.15 |  |  |  |  |
| 4 | 26 | 8.28 |  |  |  |  |
| 5 | 26 | 8.08 |  |  |  |  |
| 6 | 26 | 8.31 |  |  |  |  |
| 1 | 30 | 8.16 |  |  |  |  |
| 2 | 30 | 8.14 |  |  |  |  |
| 3 | 30 | 8.18 |  |  |  |  |
| 4 | 30 | 8.71 |  |  |  |  |
| 5 | 30 | 8.07 |  |  |  |  |
| 6 | 30 | 8.06 |  |  |  |  |
