# Supplementary material for: Living in the intertidal: desiccation and shading reduce seagrass growth, but high salinity or population of origin have no additional effect
Source: PeerJ. 2018 Jul 20;6:e5234. doi: 10.7717/peerj.5234 (PMC6055680; doi:10.7717/peerj.5234)
Supplement: Supplemental Information 3 [file peerj-06-5234-s003.docx]

**Supplementary table 2.** Nutrient levels in the water layer of experiment 2 after 28 and 61 days, analysed at the NIOZ laboratory. Methodology for ammonium and ortho-phosphate colorimetrically (Skalar and Seal autoanalyzer), using ammonium-molybdate and salicylate (Lamers et al. 1998^1^). Nitrate was determined by suphanilamide, after reduction of nitrate to nitrite in a cadmium column (Wood et al., 1967^2^). Nutrient levels showed no correlation with salinity at either date (Pearson correlations p>0.20). Averages of two replicate supply tanks are presented.

| **salinity** | **Ammonium** | **Nitrate** | **Phosphate** |
| --- | --- | --- | --- |
|  | **µmol/l** | **µmol/l** | **µmol/l** |
| **28 days** |  |  |  |
| 25 | 1.92 | 0.385 | 0.27 |
| 30 | 3.91 | 0.435 | 0.33 |
| 35 | 1.52 | 0.335 | 0.29 |
| **61 days** |  |  |  |
| 25 | 0.68 | 0 | 0.24 |
| 30 | 1.13 | 0.065 | 0.28 |
| 35 | 0.67 | 0 | 0.38 |

^1^Lamers LPM, Tomassen HBM, Roelofs JGM (1998) Sulfate-induced eutrophication and phytotoxicity in freshwater wetlands. Environmental Science & Technology 32:199-205

^2^Wood ED, Armstrong FAJ, Richards FA (1967). Determinations of nitrate in sea water by Cadmium-Copper redution to nitrite. Journal of the Marine Biological Association of the United Kingdom 47: 23-31
